# Supplementary figures and images for: Nurturing 21st century physician knowledge, skills and attitudes with medical home innovations: the Wright Center for Graduate Medical Education teaching health center curriculum experience
Source: PeerJ. 2015 Feb 10;3:e766. doi: 10.7717/peerj.766 (PMC4327443; doi:10.7717/peerj.766)

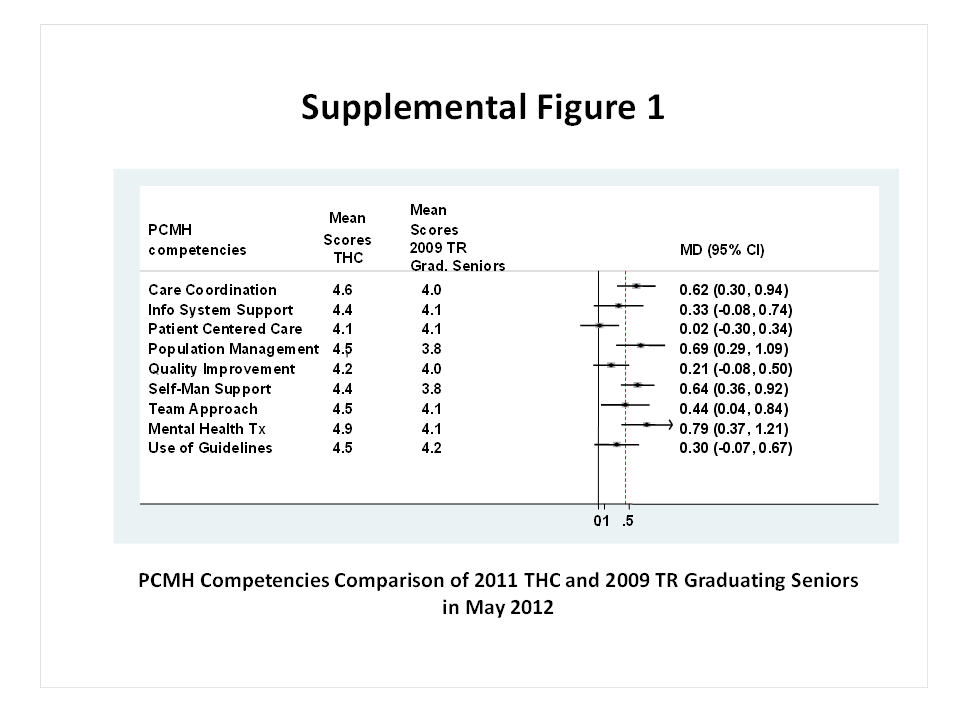

Supplement: Figure S1 — KSA comparison of 2011 THC and 2009 TR graduating seniors in May 2012. [file peerj-03-766-s001.png]

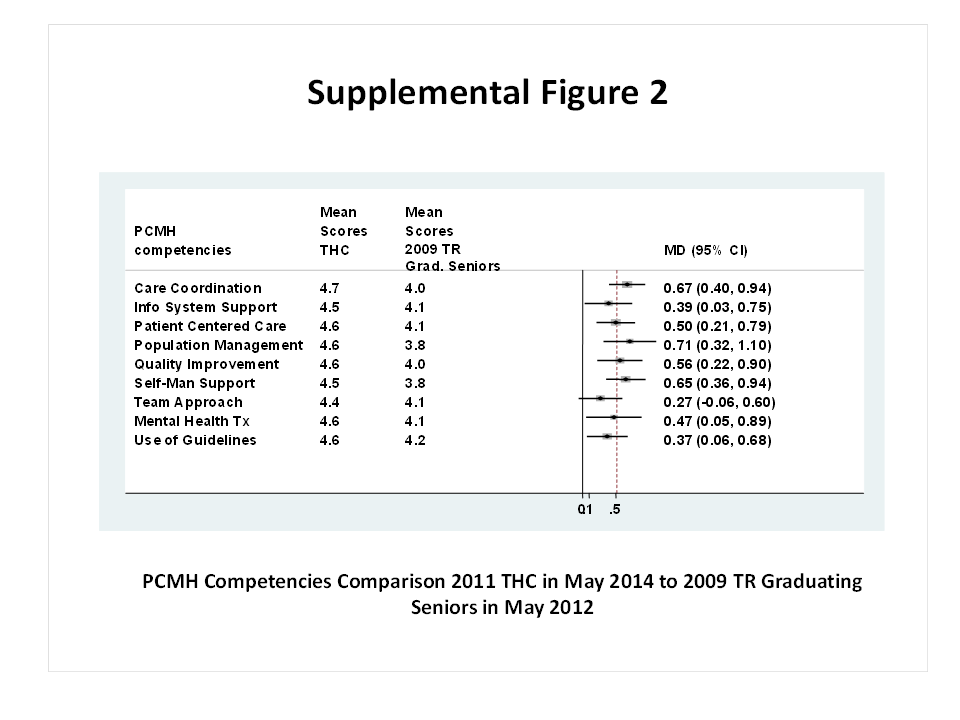

Supplement: Figure S2 — PCMH competencies comparison 2011 THC in May 2014 to 2009 TR graduating seniors in May 2012. [file peerj-03-766-s002.png]

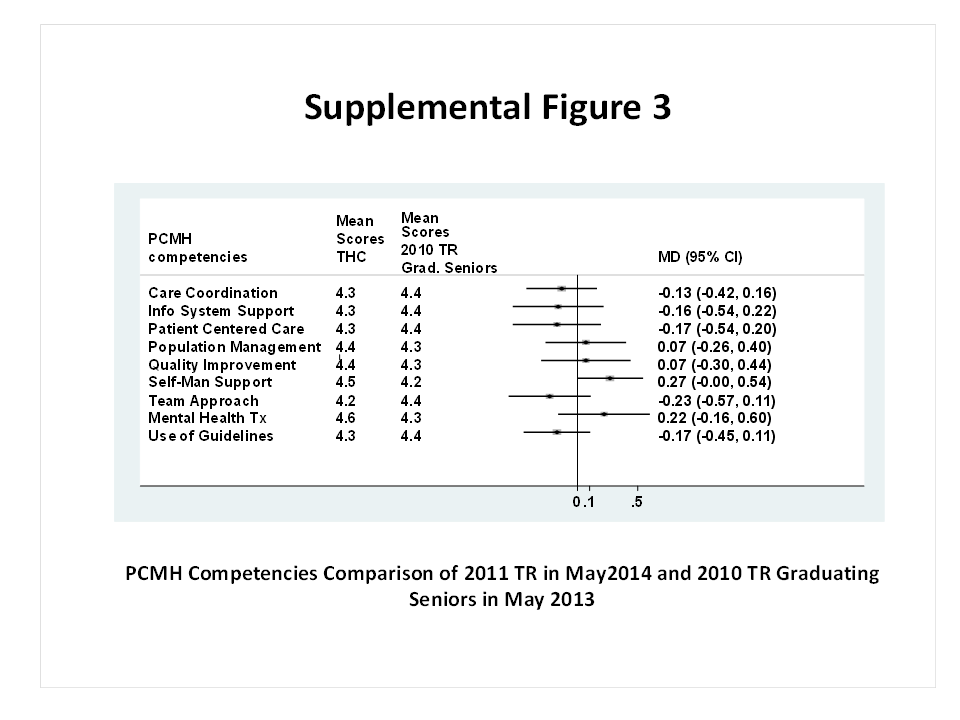

Supplement: Figure S3 — KSA comparison of 2011 TR in May 2014 and 2010 TR graduating seniors in May 2013. [file peerj-03-766-s003.png]

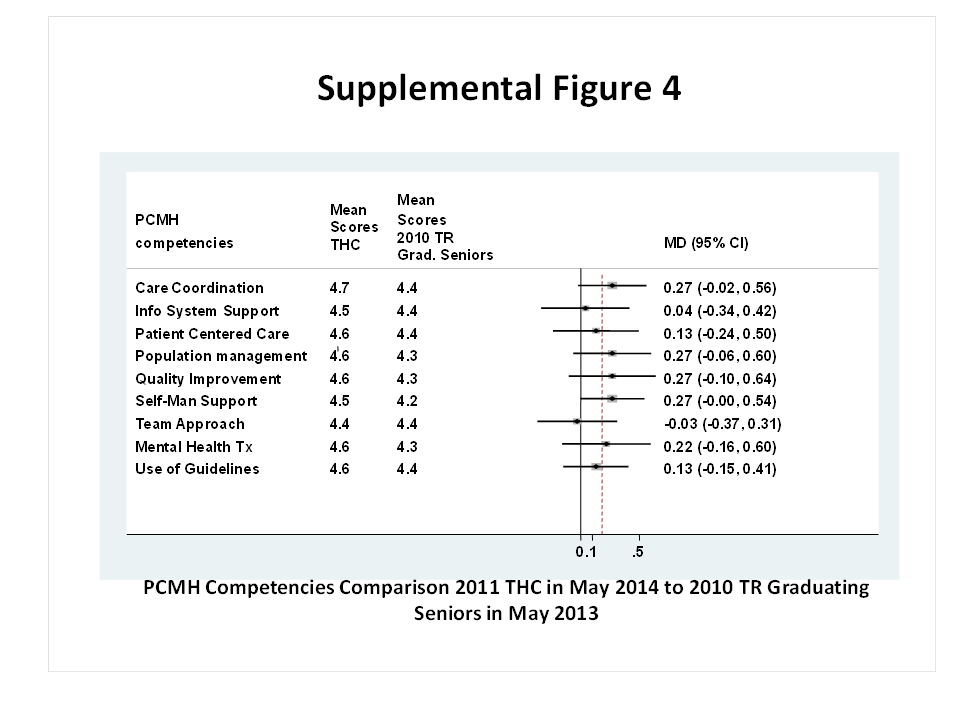

Supplement: Figure S4 — KSA comparison 2011 THC in May 2014 to 2010 TR graduating seniors in May 2013. [file peerj-03-766-s008.png]
