# Supplementary material for: Nurturing 21st century physician knowledge, skills and attitudes with medical home innovations: the Wright Center for Graduate Medical Education teaching health center curriculum experience
Source: PeerJ. 2015 Feb 10;3:e766. doi: 10.7717/peerj.766 (PMC4327443; doi:10.7717/peerj.766)
Supplement: Table S1 — May 2012 PCMH Competencies Comparison of 2011 THC Residents and 2009 TR Graduating Seniors. All competencies except Patient Centered Care improved. [file peerj-03-766-s004.docx]

**Supplemental Table 1**

| **Competency** | **2011 THC**  **May 2012 n=12** | **2009 TR Graduating Seniors**  **May 2012 n=10** | **P value** |
| --- | --- | --- | --- |
| Care Coordination | 4.7 (4.3 – 5.0) | 4.0 (3.5 – 4.5) | 0.031 |
| Info System Support | 4.4 (4.0 – 4.7) | 4.1 (3.5 – 4.6) | 0.201 |
| Patient Centered Care | 4.1 (3.7 – 4.5) | 4.1 (3.5 – 4.5) | 0.988 |
| Population Management | 4.6 (4.1 – 4.9) | 3.9 (3.5 – 4.3) | 0.022 |
| Quality Improvement | 4.3 (3.9 – 4.7) | 4.0 (3.5 – 4.5) | 0.177 |
| Self-Man Support | 4.5 (4.1 – 4.8) | 3.9 (3.5 – 4.5) | 0.038 |
| Team Approach | 4.6 (4.3 – 5.0) | 4.1 (3.6 – 4.3) | 0.046 |
| Mental Health Tx | 4.9 (4.5 – 5.0) | 4.1 (3.5 – 4.5) | 0.039 |
| Use of Guidelines | 4.5 (4.2 – 4.9) | 4.2 (3.6 – 4.5) | 0.311 |
