# Supplementary material for: Nurturing 21st century physician knowledge, skills and attitudes with medical home innovations: the Wright Center for Graduate Medical Education teaching health center curriculum experience
Source: PeerJ. 2015 Feb 10;3:e766. doi: 10.7717/peerj.766 (PMC4327443; doi:10.7717/peerj.766)
Supplement: Table S2 — May of Graduating Year PCMH Competencies Comparison of 2011 THC (in May 2014) and 2009 TR Graduating Seniors (in May 2012) and 2010 TR Graduating Seniors (in May 2013). [file peerj-03-766-s005.docx]

**Supplemental Table 2**

| **Competency** | **2011 THC**  **May 2014 n=11** | **2009 TR Graduating Seniors**  **May 2012 n=10** | **P value** | **2010 TR Graduating Seniors**  **May 2013 n=12** | **P-value** |
| --- | --- | --- | --- | --- | --- |
| Care Coordination | 4.7 (4.4 – 4.9) | 4.0 (3.5 – 4.5) | 0.029 | 4.5 (3.7 – 4.6) | 0.151 |
| Info System Support | 4.5 (4.2 – 4.8) | 4.1 (3.5 – 4.6) | 0.165 | 4.5 (3.6 – 4.7) | 0.317 |
| Patient Centered Care | 4.6 (4.3 – 4.9) | 4.1 (3.5 – 4.5) | 0.041 | 4.5 (3.8 – 4.6) | 0.277 |
| Population Management | 4.6 (4.3 – 4.9) | 3.9 (3.5 – 4.3) | 0.031 | 4.3 (3.6 – 4.6) | 0.068 |
| Quality Improvement | 4.6 (4.3 – 4.9) | 4.0 (3.5 – 4.5) | 0.049 | 4.3 (3.5 – 4.8) | 0.089 |
| Self-Man Support | 4.5 (4.2 – 4.8) | 3.9 (3.5 – 4.5) | 0.038 | 4.3 (4.0 – 4.7) | 0.199 |
| Team Approach | 4.4 (4.0 – 4.7) | 4.1 (3.6 – 4.3) | 0.199 | 4.4 (3.5 – 4.6) | 0.156 |
| Mental Health Tx | 4.6 (4.3 – 4.9) | 4.1 (3.5 – 4.5) | 0.115 | 4.4 (4.0 – 4.8) | 0.215 |
| Use of Guidelines | 4.6 (4.3 – 4.9) | 4.2 (3.6 – 4.5) | 0.131 | 4.5 (3.7 – 4.7) | 0.114 |
