# Supplementary material for: Nurturing 21st century physician knowledge, skills and attitudes with medical home innovations: the Wright Center for Graduate Medical Education teaching health center curriculum experience
Source: PeerJ. 2015 Feb 10;3:e766. doi: 10.7717/peerj.766 (PMC4327443; doi:10.7717/peerj.766)
Supplement: Table S5 — ACGME core competencies mean scores of 2011 TR residents comparisons to baseline. [file peerj-03-766-s009.docx]

**Supplemental Table 5**

| ACGME competencies | December 2011 | June 2012 | ^a^P value | December 2012 | ^b^P value | June 2014 | ^c^P value |
| --- | --- | --- | --- | --- | --- | --- | --- |
| Interpersonal and communication skills | 4.1 (3.9 – 4.4) | 4.1 (3.8 – 4.3) | 0.599 | 4.3 (4.0 – 4.6) | 0.042 | 4.4 (4.1 – 4.6) | 0.015 |
| Medical knowledge | 3.9 (3.6 – 4.3) | 4.1 (3.9 – 4.3) | 0.339 | 4.3 (4.0 – 4.6) | 0.004 | 4.4 (4.0 – 4.6) | 0.001 |
| Patient care and procedural skills | 3.8 (3.5 – 4.1) | 4.0 (3.9 – 4.2) | 0.002 | 4.3 (4.1 – 4.6) | <0.001 | 4.4 (4.0 – 4.6) | <0.001 |
| Practice based learning | 3.9 (3.7 – 4.1) | 4.0 (3.9 – 4.2) | 0.018 | 4.3 (4.0 – 4.5) | <0.001 | 4.4 (4.0 – 4.6) | <0.001 |
| Professionalism | 4.2 (4.0 – 4.4) | 4.2 (4.0 – 4.3) | 0.375 | 4.5 (4.2 – 4.7) | 0.002 | 4.4 (4.1 – 4.7) | 0.018 |
| System based skills | 3.9 (3.7 – 4.1) | 3.9 (3.8 – 4.1) | 0.131 | 4.3 (3.9 – 4.5) | <0.001 | 4.3 (4.0 – 4.6) | <0.001 |

**^a^December 2011 compared to June 2012**

**^b^December 2011 compared to December 2012**

**^c^December 2011 compared to June 2014**
