# Supplementary material for: Nurturing 21st century physician knowledge, skills and attitudes with medical home innovations: the Wright Center for Graduate Medical Education teaching health center curriculum experience
Source: PeerJ. 2015 Feb 10;3:e766. doi: 10.7717/peerj.766 (PMC4327443; doi:10.7717/peerj.766)
Supplement: Table S6 — Comparison between THC and TR 2011 residents with regards to ACGME core competency scores. [file peerj-03-766-s010.docx]

**Supplemental Table 6**

|  | December 2011 | | | June 2012 | | |
| --- | --- | --- | --- | --- | --- | --- |
| ACGME competencies | THC | TR | P value | THC | TR | P value |
| Interpersonal and communication skills | 4.1 (3.6 – 4.6) | 4.1 (3.9 – 4.4) | 0.951 | 4.1 (3.7 – 4.4) | 4.1 (3.8 – 4.3) | 0.251 |
| Medical knowledge | 3.9 (3.4 – 4.4) | 3.9 (3.6 – 4.3) | 0.218 | 4.0 (3.5 – 4.3) | 4.1 (3.9 – 4.3) | 0.277 |
| Patient care and procedural skills | 3.8 (3.4 – 4.2) | 3.8 (3.5 – 4.1) | 0.498 | 3.9 (3.8 – 4.2) | 4.0 (3.9 – 4.2) | 0.251 |
| Practice based learning | 3.8 (3.5 – 4.1) | 3.9 (3.7 – 4.1) | 0.498 | 3.9 (3.6 – 4.1) | 4.0 (3.9 – 4.2) | 0.072 |
| Professionalism | 4.1 (3.6 – 4.3) | 4.2 (4.0 – 4.4) | 0.325 | 4.1 (3.7 – 4.3) | 4.2 (4.0 – 4.3) | 0.077 |
| System based skills | 3.8 (3.5 – 4.1) | 3.9 (3.7 – 4.1) | 0.176 | 3.9 (3.5 – 4.1) | 3.9 (3.8 – 4.1) | 0.032 |
|  | December 2012 | | | June 2014 | | |
| ACGME competencies | THC | TR | P value | THC | TR | P value |
| Interpersonal and communication skills | 4.2 (3.9 – 4.4) | 4.3 (4.0 – 4.6) | 0.148 | 4.3 (3.8 – 4.6) | 4.4 (4.1 – 4.6) | 0.258 |
| Medical knowledge | 4.1 (3.6 – 4.5) | 4.3 (4.0 – 4.6) | 0.106 | 4.2 (3.6 – 4.6) | 4.4 (4.0 – 4.6) | 0.223 |
| Patient care and procedural skills | 4.1 (3.8 – 4.4) | 4.3 (4.1 – 4.6) | 0.049 | 4.2 (3.7 – 4.6) | 4.4 (4.0 – 4.6) | 0.202 |
| Practice based learning | 4.1 (3.6 – 4.3) | 4.3 (4.0 – 4.5) | 0.083 | 4.2 (3.7 – 4.6) | 4.4 (4.0 – 4.6) | 0.192 |
| Professionalism | 4.3 (4.0 – 4.5) | 4.5 (4.2 – 4.7) | 0.013 | 4.3 (3.8 – 4.6) | 4.4 (4.1 – 4.7) | 0.354 |
| System based skills | 4.1 (3.6 – 4.3) | 4.3 (3.9 – 4.5) | 0.060 | 4.2 (3.7 – 4.6) | 4.3 (4.0 – 4.6) | 0.324 |
