# Supplementary material for: Nurturing 21st century physician knowledge, skills and attitudes with medical home innovations: the Wright Center for Graduate Medical Education teaching health center curriculum experience
Source: PeerJ. 2015 Feb 10;3:e766. doi: 10.7717/peerj.766 (PMC4327443; doi:10.7717/peerj.766)
Supplement: Table S7 — Mapping of ACGME competencies to PCMH competencies (KSA). [file peerj-03-766-s011.docx]

**Supplemental Table 7**

| PCMH competencies (KSA) | ACGME competencies |
| --- | --- |
| Care coordination | - Practice based learning - Inter-personal and communication skills - Systems-Based Practices |
| Information system support | - System based skills |
| Patient centered care | - Patient care and procedural skills - Professionalism - Interpersonal communication skills - Practice-based Learning & Improvement |
| Population management | - Interpersonal and communication skills - Practice based learning - System based Practices - Medical Knowledge |
| Quality improvement | - Practice based learning & Improvement - System based skills |
| Self management support | - Medical knowledge - Inter-personal and communication skills - Patient Care - Systems-based Practices |
| Team approach | - System based skills - Practice based learning & Improvement - Professionalism - Interpersonal & Communication Skills |
| Treatment of mental health issues | - Professionalism - Inter-personal and communication skills - Medical Knowledge - Patient Care |
| Use of guidelines | - Medical knowledge - Practice-Based Learning & Improvement - Systems-Based Practices - Patient Care |
