# Supplementary material for: Nurturing 21st century physician knowledge, skills and attitudes with medical home innovations: the Wright Center for Graduate Medical Education teaching health center curriculum experience
Source: PeerJ. 2015 Feb 10;3:e766. doi: 10.7717/peerj.766 (PMC4327443; doi:10.7717/peerj.766)
Supplement: Table S8 — Longitudinal KSA mapped ACGME competencies 2011 THC residents comparisons to baseline. [file peerj-03-766-s012.docx]

**Supplemental Table 8**

| KSA mapped to ACGME competencies | December 2011 | June 2012 | ^a^P value | December 2012 | ^b^P value | June 2014 | ^c^P value |
| --- | --- | --- | --- | --- | --- | --- | --- |
| Care coordination   - Practice based learning - Inter-personal and communication skills - Systems-Based Practices | 4.1 (3.6 – 4.6) | 4.3 (4.0 – 4.6) | 0.039 | 4.4 (4.1 – 4.7) | 0.033 | 4.4 (3.8 – 4.6) | 0.038 |
| Information system support   - System based skills | 3.9 (3.4 – 4.4) | 4.2 (3.8 – 4.5) | 0.041 | 4.1 (3.6 – 4.5) | 0.003 | 4.2 (3.6 – 4.6) | 0.008 |
| Patient centered care   - Patient care and procedural skills - Professionalism - Interpersonal communication skills - Practice-based Learning & Improvement | 3.8 (3.4 – 4.2) | 4.3 (4.0 – 4.5) | 0.037 | 4.1 (3.8 – 4.4) | 0.012 | 4.2 (3.7 – 4.6) | 0.001 |
| Population management   - Interpersonal and communication skills - Practice based learning - System based Practices - Medical Knowledge | 3.8 (3.5 – 4.1) | 4.1 (3.9 – 4.4) | 0.051 | 4.1 (3.6 – 4.3) | 0.003 | 4.2 (3.7 – 4.6) | 0.002 |
| Quality Improvement   - Practice based learning - System based skills | 4.1 (3.6 – 4.3) | 4.1 (3.7 – 4.3) | 1.000 | 4.3 (4.0 – 4.5) | 0.012 | 4.3 (3.8 – 4.6) | 0.004 |
| Self management support   - Medical knowledge - Inter-personal and communication skills - Patient Care - Systems-based Practices | 3.8 (3.5 – 4.1) | 3.9 (3.5 – 4.1) | 0.3001 | 4.1 (3.6 – 4.3) | 0.003 | 4.2 (3.7 – 4.6) | <0.001 |
| Team approach   - System based skills - Practice based learning & Improvement - Professionalism - Interpersonal & Communication Skills | 4.7 (4.1 – 5.0) | 4.6 (4.0 – 4.9) | 0.806 | 4.6 (4.0 – 5.0) | 0.803 | 4.6 (4.0 – 4.8) | 0.156 |
| Treatment of Mental Health issues   - Professionalism - Inter-personal and communication skills - Medical Knowledge - Patient Care | 4.3 (4.0 – 4.7) | 4.4 (3.6 – 5.0) | 0.586 | 4.1 (3.4 – 4.5) | 0.526 | 4.5 (3.8 – 5.0) | 0.280 |
| Use of Guidelines   - Medical knowledge - Practice-Based Learning & Improvement - Systems-Based Practices - Patient Care | 4.1 (3.3 – 5.0) | 4.0 (3.3 – 4.6) | 0.933 | 4.6 (4.0 – 5.0) | 0.025 | 4.4 (4.0 – 4.9) | 0.028 |

**^a^December 2011 compared to June 2012**

**^b^December 2011 compared to December 2012**

**^c^December 2011 compared to June 2014**
