# Supplementary material for: Nurturing 21st century physician knowledge, skills and attitudes with medical home innovations: the Wright Center for Graduate Medical Education teaching health center curriculum experience
Source: PeerJ. 2015 Feb 10;3:e766. doi: 10.7717/peerj.766 (PMC4327443; doi:10.7717/peerj.766)
Supplement: Table S10 — Comparison between THC and TR 2011 residents with regards to KSA mapped ACGME competencies. [file peerj-03-766-s014.docx]

**Supplemental Table 10**

|  | December 2011 | | | June 2012 | | |
| --- | --- | --- | --- | --- | --- | --- |
| PCMH competencies mapped to ACGME competencies | THC | TR | P value | THC | TR | P value |
| Care coordination   - Practice based learning - Inter-personal and communication skills   Systems-Based Practices | 4.1 (3.6 – 4.6) | 4.1 (3.9 – 4.4) | 0.951 | 4.3 (4.0 – 4.6) | 4.3 (3.7 – 4.4) | 0.115 |
| Information system support  System based skills | 3.9 (3.4 – 4.4) | 3.9 (3.6 – 4.3) | 0.218 | 4.3 (3.8 – 4.5) | 4.2 (3.5 – 4.3) | 0.259 |
| Patient centered care   - Patient care and procedural skills - Professionalism - Interpersonal communication skills   Practice-based Learning & Improvement | 3.8 (3.4 – 4.2) | 3.8 (3.5 – 4.1) | 0.498 | 4.2 (4.0 – 4.3) | 4.1 (3.8 – 4.2) | 0.311 |
| Population management   - Interpersonal and communication skills - Practice based learning - System based Practices   Medical Knowledge | 3.8 (3.5 – 4.1) | 3.9 (3.7 – 4.1) | 0.498 | 4.0 (3.7 – 4.2) | 3.9 (3.6 – 4.1) | 0.327 |
| Quality Improvement   - Practice based learning   System based skills | 4.1 (3.6 – 4.3) | 4.2 (4.0 – 4.4) | 0.325 | 4.1 (3.7 – 4.3) | 4.1 (3.7 – 4.3) | 0.333 |
| Self management support   - Medical knowledge - Inter-personal and communication skills - Patient Care   Systems-based Practices | 3.8 (3.5 – 4.1) | 3.9 (3.7 – 4.1) | 0.176 | 3.9 (3.5 – 4.1) | 3.9 (3.5 – 4.1) | 0.710 |
| Team approach   - System based skills - Practice based learning & Improvement - Professionalism   Interpersonal & Communication Skills | 4.7 (4.1 – 5.0) | 4.6 (4.4 – 4.8) | 0.206 | 4.6 (4.0 – 4.9) | 4.6 (4.0 – 4.9) | 0.882 |
| Treatment of Mental Health issues   - Professionalism - Inter-personal and communication skills - Medical Knowledge   Patient Care | 4.3 (4.0 – 4.7) | 4.0 (3.5 – 5.0) | 0.121 | 4.4 (3.6 – 5.0) | 4.4 (3.6 – 5.0) | 0.441 |
| Use of Guidelines   - Medical knowledge - Practice-Based Learning & Improvement - Systems-Based Practices   Patient Care | 4.1 (3.3 – 5.0) | 4.2 (3.6 – 4.8) | 0.341 | 4.0 (3.3 – 4.6) | 4.0 (3.3 – 4.6) | 0.532 |
|  | December 2012 | | | June 2014 | | |
| PCMH competencies mapped to ACGME competencies | THC | TR | P value | THC | TR | P value |
| Care coordination   - Practice based learning - Inter-personal and communication skills   Systems-Based Practices | 4.4 (4.1 – 4.7) | 4.3 (4.0 – 4.6) | 0.130 | 4.4 (3.8 – 4.6) | 4.4 (4.1 – 4.6) | 0.258 |
| Information system support  System based skills | 4.3 (3.6 – 4.5) | 4.2 (4.0 – 4.6) | 0.117 | 4.2 (3.6 – 4.6) | 4.4 (4.0 – 4.6) | 0.223 |
| Patient centered care   - Patient care and procedural skills - Professionalism - Interpersonal communication skills   Practice-based Learning & Improvement | 4.1 (3.8 – 4.4) | 4.3 (4.1 – 4.6) | 0.071 | 4.2 (3.7 – 4.6) | 4.4 (4.0 – 4.6) | 0.202 |
| Population management   - Interpersonal and communication skills - Practice based learning - System based Practices   Medical Knowledge | 4.2 (3.6 – 4.3) | 4.3 (4.0 – 4.5) | 0.117 | 4.2 (3.7 – 4.6) | 4.4 (4.0 – 4.6) | 0.192 |
| Quality Improvement   - Practice based learning   System based skills | 4.3 (4.0 – 4.5) | 4.3 (4.2 – 4.7) | 0.177 | 4.3 (3.8 – 4.6) | 4.4 (4.1 – 4.7) | 0.354 |
| Self management support   - Medical knowledge - Inter-personal and communication skills - Patient Care   Systems-based Practices | 4.1 (3.6 – 4.3) | 4.3 (3.9 – 4.5) | 0.100 | 4.2 (3.7 – 4.6) | 4.3 (4.0 – 4.6) | 0.324 |
| Team approach   - System based skills - Practice based learning & Improvement - Professionalism   Interpersonal & Communication Skills | 4.6 (4.0 – 5.0) | 4.9 (4.3 – 5.0) | 0.164 | 4.6 (4.0 – 4.8) | 4.6 (4.0 – 5.0) | 0.183 |
| Treatment of Mental Health issues   - Professionalism - Inter-personal and communication skills - Medical Knowledge   Patient Care | 4.1 (3.4 – 4.5) | 4.5 (4.1 – 4.6) | 0.157 | 4.5 (3.8 – 5.0) | 4.5 (4.0 – 5.0) | 0.717 |
| Use of Guidelines   - Medical knowledge - Practice-Based Learning & Improvement - Systems-Based Practices   Patient Care | 4.6 (4.0 – 5.0) | 4.7 (4.0 – 5.0) | 0.061 | 4.4 (4.0 – 4.9) | 4.5 (4.0 – 5.0) | 0.438 |
