# Supplementary material for: Nurturing 21st century physician knowledge, skills and attitudes with medical home innovations: the Wright Center for Graduate Medical Education teaching health center curriculum experience
Source: PeerJ. 2015 Feb 10;3:e766. doi: 10.7717/peerj.766 (PMC4327443; doi:10.7717/peerj.766)
Supplement: Table S11 — Correlation between KSA scores from sruvey and KSA mapped ACGME competency mean scores in Deccember 2012 (THC residents). [file peerj-03-766-s015.docx]

**Supplemental Table 11**

| PCMH competencies mapped to ACGME competencies | Care coordination | Information system support | Patient centered care | Population management | Quality improvement | Self management support | Team approach | Treatment of mental health issues | Use of guidelines |
| --- | --- | --- | --- | --- | --- | --- | --- | --- | --- |
| Care coordination   - Practice based learning - Inter-personal and communication skills   Systems-Based Practices | 0.4 |  |  |  |  |  |  |  |  |
| Information system support  System based skills |  | ^a^0.6 |  |  |  |  |  |  |  |
| Patient centered care   - Patient care and procedural skills - Professionalism - Interpersonal communication skills   Practice-based Learning & Improvement |  |  | ^a^0.6 |  |  |  |  |  |  |
| Population management   - Interpersonal and communication skills - Practice based learning - System based Practices   Medical Knowledge |  |  |  | 0.4 |  |  |  |  |  |
| Quality Improvement   - Practice based learning   System based skills |  |  |  |  | -0.1 |  |  |  |  |
| Self management support   - Medical knowledge - Inter-personal and communication skills - Patient Care   Systems-based Practices |  |  |  |  |  | 0.1 |  |  |  |
| Team approach   - System based skills - Practice based learning & Improvement - Professionalism   Interpersonal & Communication Skills |  |  |  |  |  |  | 0.5 |  |  |
| Treatment of Mental Health issues   - Professionalism - Inter-personal and communication skills - Medical Knowledge   Patient Care |  |  |  |  |  |  |  | -0.3 |  |
| Use of Guidelines   - Medical knowledge - Practice-Based Learning & Improvement - Systems-Based Practices   Patient Care |  |  |  |  |  |  |  |  | 0.2 |

**^a^: denotes statistically significant difference**
