# Supplementary material for: Nurturing 21st century physician knowledge, skills and attitudes with medical home innovations: the Wright Center for Graduate Medical Education teaching health center curriculum experience
Source: PeerJ. 2015 Feb 10;3:e766. doi: 10.7717/peerj.766 (PMC4327443; doi:10.7717/peerj.766)
Supplement: Analysis S1 — Detailed analysis assessing improvement in ACGME core competencies in our study residents and their correlation with KSA. [file peerj-03-766-s019.docx]

**Supplemental analysis**

**Objective:** In the main manuscript we report that implementation of PCMH curricular innovations at the Wright Center for Graduate Medical Education (WCGME), was associated with a consistent improvement in our Internal Medicine (IM) residents’ KSA. In the analysis reported below we sought to assess if these improvements in KSA also paralleled objective ACGME core competency based assessments.

**Study population:** Twenty four Internal Medicine residents (2011 THC residents: 12 and 2011 TR residents: 12)

**Study tool**: ACGME core competencies have been reported and explained in detail by Green et. al. ([J Grad Med Educ.](http://www.ncbi.nlm.nih.gov/pubmed/?term=Charting+the+Road+to+Competence%3ADevelopmental+Milestones+for+Internal) 2009;1(1):5-20) (reference 1 in this supplemental file). They comprise of 6 competencies:

1. Inter-personal communication skills
2. Medical Knowledge
3. Patient care and procedural skills
4. Practice based learning and improvement
5. Professionalism
6. System based skills

The study residents were assessed based on these 6 competencies at the following points in time during the 3 years of their residency training: 1) December 2011 (baseline assessment); pools all assessments completed between July and December 2011 for both 2011 THC and 2011 TR residents, 2) June 2012: pools all assessments completed between January and June 2012; corresponds to the period of focal implementation of PCMH curricular innovations in the WCGME applied only to 2011 THC residents, 3) December 2012: pools all assessments completed between July and December 2012; corresponds to period of global implementation of PCMH curricular innovations applied on all IM residents (2011 THC residents and 2011 TR residents), 4) June 2014: pools all assessments completed between January and June 2014; corresponds to the time point when the IM residents completed training and graduated in June 2014. All assessments were completed by attending physicians, peers, support staff and extenders responsible for the residents’ training and supervision. The ACGME core competency based assessments were graded between 0 – 5, where 0 represented poor performance and 5 represented superior performance.

**Statistical analysis**: mean scores for each of the ACGME core competencies mentioned about were calculated. Since the scores were non-parametrically distributed, continuous variables were compared using Wilcoxon rank sum test and categorical variables were compared between groups using chi-squared test. P < 0.05 was considered statistically significant. All analyses were performed using STATA 11 statistical software. Mean scores for ACGME core competencies were compared longitudinally for both 2011 THC and TR residents over 3 years of training at the above defined time points, with comparisons to baseline scores. Between groups cross-sectional comparisons of mean individual ACGME core competency scores at the defined time points were done for THC and TR residents.

Then, the 6 ACGME competencies mentioned above were mapped with each of the 9 KSA mentioned in the main manuscript. This mapping was performed to assess if these ACGME core competency scores correlate with the KSA survey mean scores cross-sectionally at the above mentioned time points. This mapping was subjective as there is no objective report in the literature or by the ACGME, to the best of our knowledge, which had mapped the ACGME core competencies to the KSA. Then the mean scores of the ACGME core competencies mapped to each of the individual KSA were pooled together, averaged and reported as the KSA mapped ACGME competency mean scores and were compared longitudinally for both 2011 THC and TR residents over 3 years of training at the above defined time points, with comparisons to baseline scores. Similarly between groups cross-sectional comparisons of KSA mapped ACGME competency mean scores at the defined time points were done for THC and TR residents.

Finally, the KSA mapped ACGME competency mean scores were correlated with the cross-sectional KSA survey mean scores (from the main manuscript) at the above defined time points. Pearson’s correlation co-efficient ‘r’ was used to assess the correlation. An ‘r’ value between -1 to 0 was considered negative correlation while a value between 0 and +1 was considered positive correlation.

**Results**: When compared to baseline (December 2011), the THC residents scored similar in all ACGME core competencies by June 2012 but improved in many competencies by December 2012 and sustained these improvements until June 2014 (Supplemental Table 4). The TR residents, when compared to baseline (December 2011) showed improvements as early as June 2012 in some competencies, consistently improved in many other competencies by December 2012 and sustained these improvements until June 2014 (Supplemental Table 5). Cross-sectional comparisons showed that the ACGME competency scores were similar at baseline (December 2011) when THC residents were compared to TR residents (Supplemental Table 6). In June 2012, the mean ACGME core competency scores were similar between the THC and TR residents in most milestones except system based skills (Supplemental Table 6) where TR residents score better. By December 2012, THC and TR residents scored similar in most ACGME core competencies except in patient care, procedural skills, and professionalism where TR residents scored higher than the THC residents. However, by June 2014 the time of training completion, mean scores for all 6 ACGME core competencies were similar when THC residents were compared with TR residents (Supplemental Table 6).

Mapping between the ACGME competencies and KSA are detailed in Supplemental Table 7. Compared to baseline (December 2011), THC residents showed improvement in a few KSA mapped ACGME competency mean scores (Supplemental Table 8) by June 2012. By December 2012, they improved on several others and sustained the improvement until June 2014 (Supplemental Table 8). The TR residents scored similar to baseline on KSA mapped ACGME competency mean scores by June 2012, but improved in several competencies by December 2012 and maintained the improvement until June 2014 (Supplemental Table 9). Cross sectional comparisons between THC and TR residents showed similar scores at defined time points for KSA mapped ACGME competency mean scores (Supplemental Table 10). Correlation analysis assessing the correlation between KSA survey mean scores and KSA mapped ACGME competency mean scores showed positive correlation (r between 0 to +1) in most comparisons with some reaching statistical significance for both THC and TR residents by December 2012 and by June 2014 (Supplemental Table 11, Supplemental Table 12, Supplemental Table 13 and Supplemental Table 14).

**Interpretation**: Our analysis shows that 2011 THC and TR residents showed improvement in ACGME core competencies by December 2012. These improvements in scores were sustained by both THC and TR residents until completion of their training. There were positive correlations between KSA survey mean scores and KSA mapped ACGME competency mean scores for most comparisons in both 2011 THC and TR residents by December 2012 and sustained until June 2014.

**Limitations of these analyses**: The mapping between KSA survey scores and KSA mapped ACGME competency mean scores were subjective as there is a lack of objective mapping reported in the literature. Also, the strengths of correlation were weak, though positive, for some of the competencies.

**Reference**:

1. [Green ML](http://www.ncbi.nlm.nih.gov/pubmed/?term=Green%20ML%5BAuthor%5D&cauthor=true&cauthor_uid=21975701), [Aagaard EM](http://www.ncbi.nlm.nih.gov/pubmed/?term=Aagaard%20EM%5BAuthor%5D&cauthor=true&cauthor_uid=21975701), [Caverzagie KJ](http://www.ncbi.nlm.nih.gov/pubmed/?term=Caverzagie%20KJ%5BAuthor%5D&cauthor=true&cauthor_uid=21975701), [Chick DA](http://www.ncbi.nlm.nih.gov/pubmed/?term=Chick%20DA%5BAuthor%5D&cauthor=true&cauthor_uid=21975701), [Holmboe E](http://www.ncbi.nlm.nih.gov/pubmed/?term=Holmboe%20E%5BAuthor%5D&cauthor=true&cauthor_uid=21975701), [Kane G](http://www.ncbi.nlm.nih.gov/pubmed/?term=Kane%20G%5BAuthor%5D&cauthor=true&cauthor_uid=21975701), [Smith CD](http://www.ncbi.nlm.nih.gov/pubmed/?term=Smith%20CD%5BAuthor%5D&cauthor=true&cauthor_uid=21975701), [Iobst W](http://www.ncbi.nlm.nih.gov/pubmed/?term=Iobst%20W%5BAuthor%5D&cauthor=true&cauthor_uid=21975701). Charting the road to competence: developmental milestones for internal medicine residency training. [J Grad Med Educ.](http://www.ncbi.nlm.nih.gov/pubmed/?term=Charting+the+Road+to+Competence%3ADevelopmental+Milestones+for+Internal) 2009;1(1):5-20.
