# Supplementary material for: Nurturing 21st century physician knowledge, skills and attitudes with medical home innovations: the Wright Center for Graduate Medical Education teaching health center curriculum experience
Source: PeerJ. 2015 Feb 10;3:e766. doi: 10.7717/peerj.766 (PMC4327443; doi:10.7717/peerj.766)
Supplement: File S1 — Contains the KSA survey and the informed consent statement at the bottom of the file. [file peerj-03-766-s020.doc]

# **Patient Centered Medical Home Clinician Assessment**

***Please answer the following questions based on the procedures and approaches used by you and your immediate care team (e.g. those nurses and office staff that you work with most closely on a daily basis) to take care of your patients. Do NOT answer this on the basis of your overall organization or even the entire clinic - but based on YOUR practice team. Circle the best answer.***

**Team Approach (1, 2, 10, 15 & 17)**

**1. My approach to improving the care of my patients with chronic disease can be characterized as…**

… I see these patients and provide the … I try to keep track of my patients with …I have implemented formal systems for

services they need when they come to chronic diseases to monitor their care making sure that my patients with chronic

see me in my office. between visits, but I haven’t established disease are closely monitored, whether

formal systems for doing this. they come in for office visits or not.

1 2 3 4 5

2. I use other staff members from my practice in my care of patients with chronic disease for such things as checking with patients on their adherence and response to treatment, patient education, patient self-management support, etc…

… rarely, generally doing it all … sometimes, with specific patients. …routinely, with other staff members

myself. having clearly defined roles as part of a care

team for my patients with chronic disease.

1 2 3 4 5

**10. The care of my patients with chronic disease …**

…primarily relies on me, with …centers on me, but with some help …is a well-coordinated team effort

few other resources involved. from other resources within my practice involving a number of different people and

resources.

1 2 3 4 5

**15. Follow-up of my patients with chronic disease…**

…is largely left up to the patient …is scheduled by the front desk in …is assured by my care team, which contacts

to return as necessary. accordance with guidelines that we the patient between visits to check on adherence

have set up to the treatment plan, progress, side effects, etc.

1 2 3 4 5

17. Contact with my patients with chronic disease between office visits …

…is done by me on an as-needed …is done by me or by other care team …is done on a planned basis by me or other

basis with selected patients. members on a planned basis with trained care team members with most or all

selected patients. patients with chronic disease, using a

system with tracking and reminders.

1 2 3 4 5

**The Info System Support (3, 4, 5, 9, 16, 22, 23, 28)**

**3. A registry is a list of patients with a particular chronic disease or other condition that includes such things as patient name, contact information, date of last visit, and services that are due to be provided. Such a registry …**

…is not available in my practice, or is …is available in my practice, and I use it …is available in my practice, and I use it

available but I don’t use it. sometimes with my patients with a particular actively in tracking the care of most of my

chronic disease. patients with chronic disease.

1 2 3 4 5

**5. I use flow sheets for my patients with chronic disease to provide a guide to management and to track critical elements of care …**

….never …sometimes, with selected patients …routinely, with most or all patients

1 2 3 4 5

**9. The information systems, registries, and/or patient records that I use in my care of patients with chronic disease…**

…do not include information … include results of patient assessments …include results of patient assessments,

related to patient self-management (such as health behaviors and readiness to self-management goals developed jointly

goals. engage in self-management activities), but with the patient, and reminders for the no specific patient self-management goals. clinician to periodically follow-up and

re-evaluate the goals.

1 2 3 4 5

**16. I use flow sheets for my continuity patients to track their health maintenance and preventive care issues …**

….never …sometimes, with selected patients or …routinely, with most or all patients and

limited health maintenance issues. for most health maintenance issues.

1 2 3 4 5

**22. Information about relevant subgroups of my patients with chronic disease needing services (such as those needing labs or referrals, not returning for follow-up, etc) …**

…is not available, or is available but …can be obtained upon request, and …is provided to me routinely and is used by

I don’t use the information. I occasionally use the information. me and my team to help deliver planned

care to my patients with chronic disease.

1 2 3 4 5

**4. Reminders to clinicians of needed services for patients (either electronically or through some sort of paper tickler system) …**

…are not available in my practice, or … are available, and I use them sometimes …are used actively and regularly in the care

are available but I don’t use them in the care of my patients. of my patients by me and my care team.

1 2 3 4 5

**23. In my delivery of preventive services to my patients, I…**

…rely on my patients coming …have a system for tracking where …have a system for tracking where

in for health maintenance visits. my patients stand on preventive services my patients stand on preventive services that

so I can remind them of what they need is used to send patients reminders regarding

whenever they present for care. needed services.

1 2 3 4 5

28. My use of guidelines at the point of care to guide my decisions regarding the care of patients…

…relies on my memory regarding the …is supported by guideline-based reminders of …is supported by automated reminder systems

guidelines and what needs to be needed services for patients in a few key areas. based on guidelines for most chronic conditions accomplished for each patient. and preventive care areas and tailored to

patients’ needs and self-management goals.

1. 2 3 4 5

**The Self-Management Support (7, 8, 9, 11, 13, 18, 26)**

7. I assess the self-management needs and activities of my patients with chronic disease…

…rarely. … occasionally. … routinely. 1 2 3 4 5

**8. I provide self-management support for my patients…**

…rarely, or by distributing …by distributing materials to help …by distributing materials and providing

educational materials (such as patients develop individualized self- counseling to help patients develop

(pamphlets, or booklets) that do management plans, but without formal individualized self-management plans, and

not include any specific self- follow-up on those plans with the having members of my care team follow up with

management strategies. patients. the patients to reinforce their progress.

1 2 3 4 5

**9. The information systems, registries, and/or patient records that I use in my care of patients with chronic disease…**

…do not include information … include results of patient assessments …include results of patient assessments,

related to patient self-management (such as health behaviors and readiness to self-management goals developed jointly

goals. engage in self-management activities), but with the patient, and reminders for the no specific patient self-management goals. clinician to periodically follow-up and

re-evaluate the goals.

1 2 3 4 5

**11. Setting specific patient-centered goals for health behavior change or for issues surrounding chronic diseases….**

…is generally not done with my …occurs sporadically with selected patients, …is done collaboratively with most patients,

patients, as I set the goals for their who are highly motivated and assertive. with specific goals that are systematically

care and management. reassessed and progress documented

on the patient’s chart.

1 2 3 4 5

**26. When my patients need counseling regarding health behavior changes (such as for diet, exercise, or stopping smoking), I…**

…provide limited counseling myself, …provide limited counseling myself, plus …provide extensive counseling myself based on

with few or no other services available recommend at least some limited services goals set by the patients and/or refer them to

to assist them. that are available in the community. specific services in my practice or community that are coordinated with my care.

1 2 3 4 5

**13. I share information with my patients regarding evidence based guidelines for their chronic disease….**

…rarely or never. …as part of patient education materials …to assist patients and families in setting self-

provided to patients to help them understand management goals and tracking their own

their care. care.

1 2 3 4 5

**18. I arrange for education for my patients with chronic disease (such as diet and other diabetic education for patients with diabetes)…**

…rarely, doing most of the …for many of my patients with chronic …for most or all of my patients with

education myself. disease by referral to people in my practice chronic diseases through integrated . or the community who can provide most of the education service that coordinates with my

education needed. care through active communication.

1 2 3 4 5

The Use of Guidelines (12, 13, 28)

12. I use evidence-based guidelines for various chronic diseases.…

… rarely or never …to guide my patient care in general, but …as the template for my care of my patients

not in any formal way in my practice. with chronic disease, forming the basis for flow charts and systems used to monitor their care.

1 2 3 4 5

**13. I share information with my patients regarding evidence based guidelines for their chronic disease….**

…rarely or never. …as part of patient education materials …to assist patients and families in setting self-

provided to patients to help them understand management goals and tracking their own

their care. care.

1 2 3 4 5

28. My use of guidelines at the point of care to guide my decisions regarding the care of patients…

…relies on my memory regarding the …is supported by guideline-based reminders of …is supported by automated reminder systems

guidelines and what needs to be needed services for patients in a few key areas. based on guidelines for most chronic conditions accomplished for each patient. and preventive care areas and tailored to

patients’ needs and self-management goals.

1. 2 3 4 5

**The Quality Improvement (6, 24, 25, 35)**

**6. Feedback through performance measures regarding the care of my populations of patients with particular chronic diseases…**

…is not available, or is available but …is provided and has some influence …is routinely provided, and I use the

I don’t really use the information in the on how I practice. feedback to monitor my performance

care of my patients. and make changes in how I provide care to

my patients with chronic disease.

1 2 3 4 5

**24. When I receive feedback on my performance in the form of performance measurement data, I….**

…pay little attention to …use the data for myself to point …share the data with the rest of my practice

it. out areas that I need to work on, but as part of a process to identify and improve

with no formal process for improvement. performance.

1 2 3 4 5

**25. My level of participation in my practice’s quality improvement process can be characterized as…**

…I work on things informally …My practice has an improvement process …I am an active part of our practice’s

to improve my care, but don’t that operates sporadically, and I participate improvement process, which is very active

have a formal process. in it at times. and meets regularly.

1 2 3 4 5

**35. Considering our quality improvement processes in my practice…**

…I do not believe it is necessary to involve …I believe that patients and families can help …I actively engage patients in my practice’s

patients and families in our QI process. in enhancing my practice’s QI process and quality improvement process.

and occasionally ask for their input.

1 2 3 4 5

**The Population Management (1, 3, 5, 22, 23)**

**1. My approach to improving the care of my patients with chronic disease can be characterized as…**

… I see these patients and provide the … I try to keep track of my patients with …I have implemented formal systems for

services they need when they come to chronic diseases to monitor their care making sure that my patients with chronic

see me in my office. between visits, but I haven’t established disease are closely monitored, whether

formal systems for doing this. they come in for office visits or not.

1 2 3 4 5

**3. A registry is a list of patients with a particular chronic disease or other condition that includes such things as patient name, contact information, date of last visit, and services that are due to be provided. Such a registry …**

…is not available in my practice, or is …is available in my practice, and I use it …is available in my practice, and I use it

available but I don’t use it. sometimes with my patients with a particular actively in tracking the care of most of my

chronic disease. patients with chronic disease.

1 2 3 4 5

**5. I use flow sheets for my patients with chronic disease to provide a guide to management and to track critical elements of care …**

….never …sometimes, with selected patients …routinely, with most or all patients

1 2 3 4 5

**22. Information about relevant subgroups of my patients with chronic disease needing services (such as those needing labs or referrals, not returning for follow-up, etc) …**

…is not available, or is available but …can be obtained upon request, and …is provided to me routinely and is used by

I don’t use the information. I occasionally use the information. me and my team to help deliver planned

care to my patients with chronic disease.

1 2 3 4 5

**23. In my delivery of preventive services to my patients, I…**

…rely on my patients coming …have a system for tracking where …have a system for tracking where

in for health maintenance visits. my patients stand on preventive services my patients stand on preventive services that

so I can remind them of what they need is used to send patients reminders regarding

whenever they present for care. needed services.

1. 2 3 4 5

**The Coordination of Care (11, 14, 15, 19, 29)**

**11. Setting specific patient-centered goals for health behavior change or for issues surrounding chronic diseases….**

…is generally not done with my …occurs sporadically with selected patients, …is done collaboratively with most patients,

patients, as I set the goals for their who are highly motivated and assertive. with specific goals that are systematically

care and management. reassessed and progress documented

on the patient’s chart.

1 2 3 4 5

14. Consultation with specialists to help in taking care of my patients with chronic disease…

…is accomplished by referral to specialists …is accomplished by referral to some …is coordinated with my care through

who seldom communicate with me about specialists who communicate with me well through active and effective communication

treatment plans and patient progress. and regularly and others who don’t. with specialists in most areas of care.

1 2 3 4 5

**15. Follow-up of my patients with chronic disease…**

…is largely left up to the patient …is scheduled by the front desk in …is assured by my care team, which contacts

to return as necessary. accordance with guidelines that we the patient between visits to check on adherence

have set up to the treatment plan, progress, side effects, etc.

1 2 3 4 5

**19. In order to improve the level of care available for my patients with chronic disease…**

…I have focused on my own practice …I have sought out information regarding …I have actively worked with community

and not on community resources for community resources, but have not resources to impact the level of services

patients with chronic disease. attempted to link with those resources. available for patients with chronic disease

and to coordinate the care of my patients.

1 2 3 4 5

**29. The care plans for my patients…**

…are basically outlined in my progress …are summarized in a specific care plan …are summarized in a care plan in the chart

notes from patient visits. in the chart that is available to my staff. that includes patient goals and preferences for

treatment and is used to guide the efforts of

everyone involved with the patients’ care.

1. 2 3 4 5

**The Patient Centered Care (27, 31, 32, 33, 34)**

**27. When discussing treatment options with patients, I…**

…tell the patients my selection of the best …outline other treatment options as well as …carefully discuss the options and patient

choice for them, mentioning other options my own selection to see if they have strong preferences, jointly coming to a consensus

as I think necessary. feelings about the choice. regarding the best selection for the particular

patient and situation.

1 2 3 4 5

**31. The planning of care for my patients …**

…flows from my assessment of the …is done by me, but with some discussion …is done through interactive discussions and

patient’s needs. of the patient’s specific needs and desires. goal setting with the patient and family by me

and my care team.

1 2 3 4 5

**32. In thinking about the composition of the team in my care of my patients…**

…I view the team as consisting of …I view the patient and family as part of the …I actively engage the patient and family in

health professionals only. team managing the patient’s chronic illness. setting goals and managing the patient care plan.

1 2 3 4 5

**33. In sharing clinical information with patients…**

…a paper copy of medication lists or lab/x-ray …there is a system in place through which I make …there is a web- based system for patients to

reports is provided to the patient upon request. sure that patients are provided with their clinical access their clinical information and share their

information, including lab/ray reports and medication personal health information with me or my staff.

lists.

1 2 3 4 5

**34. In order to enhance support for my patients with chronic disease…**

I do not make use of peer support groups. …I sometimes suggest that patients and families …I routinely assist patients in connecting with

find a peer support group. peer support groups in the community.

1 2 3 4 5

**Mental Health issues (21, 30, 20):**

**21. Emotional health (such as symptoms of depression or anxiety, sources of stress, family conflicts)…**

…is not routinely assessed …is assessed in my patients by …is routinely assessed in my patients

in my patients unless me when I see indicators that they are using standardized screening and monitoring

they bring up problems. having problems. protocols.

1 2 3 4 5

**30. Effective mental health counseling for my patients with mental health issues…**

…is difficult to arrange, but I can …is available by referral to mental health …is readily available and is coordinated

make a referral for patients who specialists who sometimes communicate with with my care through active and effective

seriously need it. me regarding treatment plans and patient communication with the mental health

progress. specialist.

1 2 3 4 5

**20. I use a symptom checklist with my depressed patients to monitor patient progress and change in the number or severity of depression symptoms …**

….never …sometimes, focusing on patients who …routinely, to monitor treatment response

do not seem to be improving. and to watch for relapse on stopping therapy.

1 2 3 4 5

**Name of your practice**: _____________________________________________________

**Your position in the practice *(check the best response)*:**

Clinical faculty:

    Physician ______

    PA/NP _____

    Behavioral _____

    Other _____

Non-clinical faculty _____

First year resident _____

Second year resident _____

Third year resident _____

Other _____

**How long have you worked in this practice?** _____ years and _____ months.

**Consent**

**By completing this survey I hereby consent to participate in the study. I have been explained the study details and I understand that my responses will be held confidential. I realize that I have the right to remove consent at any stage prior to publication of the study data. I whole heartedly agree to this and by completing the survey I hereby give my informed consent.**
